# N,P-Codoped Carbon Layer Coupled with MoP Nanoparticles as an Efficient Electrocatalyst for Hydrogen Evolution Reaction

**DOI:** 10.3390/ma11081316

**Published:** 2018-07-30

**Authors:** Shuai Wang, Jia Wang, Ping Li, Zexing Wu, Xien Liu

**Affiliations:** State Key Laboratory Base of Eco-chemical Engineering, College of Chemistry and Molecular Engineering, Qingdao University of Science & Technology, Qingdao 266042, China; qustwangshuai@qust.edu.cn (S.W.); 18753363755@163.com (J.W.); qustlipingest@163.com (P.L.)

**Keywords:** electrocatalysts, hydrogen evolution reaction, carbon layer, molybdenum phosphide

## Abstract

Efficient electrocatalyst plays a significant role on the development of hydrogen energy. In this work, an N,P-codoped carbon layer coupled with MoP nanoparticles (MoP/NPCs) was prepared through a facile high-temperature pyrolysis treatment. The obtained MoP/NPCs presented efficient activity for hydrogen evolution reaction (HER), with low onset potential of 90 mV, and a small Tafel slope (71 mV dec^−1^), as well as extraordinary stability in acidic electrolyte. This work provides a new facile strategy for the design and synthesis of sustainable and effective molybdenum-based electrocatalysts as alternatives to non-Pt catalysts for HER.

## 1. Introduction

As is known to all, fossil energy (i.e., oil, natural gas, coal) suffers from excessive consumption with rapidly growing economies, which results in global environmental destruction and energy crisis [1,2,3,4]. Researchers have focused on the development of efficient energy devices and green energy like fuel cells to alleviate the increasingly serious environmental pollution and depletion of energy resources [5,6,7]. With the characteristics of renewability, cleanness, and high energy-density fuel, hydrogen has been extensively studied as a promising alternative energy resource to replace non-renewable fossil fuels prepared by water electrolysis [8,9,10,11]. Generally, Pt-based materials have been recognized as the most effective electrocatalyst, as its large cathodic current densities and low overpotential for hydrogen evolution reaction (HER), plays a significant role in water electrolysis [12,13,14,15,16,17]. However, its high cost and inadequate storage cumbers its wide application. Thus, researchers have devoted a good deal of effort into developing high-activity, low-cost, and relatively abundant electrocatalysts [18,19,20,21,22,23,24].

Molybdenum phosphide (MoP) has extensively been investigated for HER, due to its similar electronic structure and excellent catalytic performance for hydrodesulfurization, which have similar reaction processes to HER [25,26,27,28]. Schaak et al. [29] synthesized amorphous MoP nanoparticles which exhibited high performance of HER in acid electrolytes. A self-supported electrocatalyst MoP@NPCNFs was developed, and it revealed remarkable catalytic activity for HER [25]. Molybdenum phosphide was also investigated for HER in both acid and alkaline electrolytes [30,31]. However, there still exists room for practical consideration of MoP, as negatively charged P sites and passive supporters can easily be formed on the surface at high temperatures, which can hinder the reaction process. Thus, it can be seen that there is a challenge to develop well-crystallized MoP nanoparticles as an efficient electrocatalyst for HER.

Herein, we develop an efficient strategy to form MoP/NPC which exhibit excellent HER performance. The prepared MoP/NPC catalyst exhibits high efficient hydrogen evolution reaction (HER) activity with a low onset potential of 90 mV, and a small Tafel slope (71 mV dec^−1^), as well as extraordinary stability in acidic electrolyte, which demonstrates a new route to prepare sustainable and effective electrocatalysts as alternatives to non-Pt catalysts for HER.

## 2. Materials and Methods

### 2.1. Synthesis of Catalysts

**Scheme 1:**
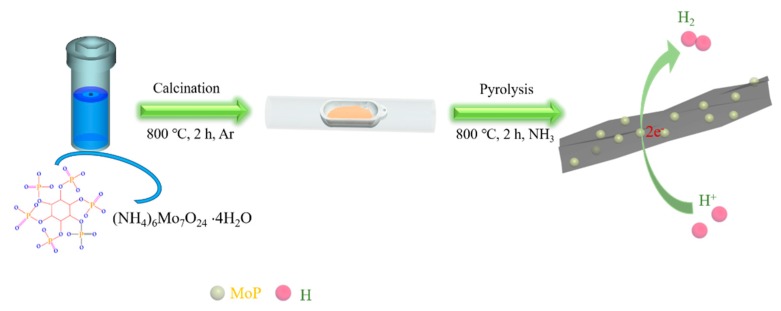
Schematic illustration for the synthesis of MoP/NPCs.

As demonstrated in Scheme 1, MoP/NPCs were synthesized via a two-step route. Primarily, 400 mg of ammonium molybdate (Aladdin, Aladdin Co. LTD, Shanghai, China) was added into 2 mL phytic acid (50 wt. %, Sigma–Aldrich, Merck KGaA, Darmstadt, Germany) to form a homogenous solution under continuous ultrasonic blending. Then, acetone (20 mL) was added to the solution to precipitate metal glue. After, the metal glue was washed with acetone. After that, the glue was transferred into a tube furnace and annealed at 800 °C for 30 min with Ar flowing at 150 mL/min and at rising rate of 5 °C/min, followed by a cooling procedure at room temperature under Ar. Here, the black products were collected and washed with 200 mL 2 M HCl under refluxing for 24 h at 95 °C. Afterwards, the acid solution was filtered, and the black powder deposited on the filter paper was adequately washed with amounts of distilled water. After being dried in a vacuum at 80 °C for 2 h, the above products were then annealed at 800 °C for 1 h with Ar and NH_3_, with each gas flowing at 500 mL/min, and then cooled to room temperature at Ar atmosphere. The resulting catalysts MoP/NPCs were then collected. For comparison, H_2_ was used as a replacement for NH_3_ at the end of the process, while the other steps remained consistent, after which MoPON/C catalyst was obtained.

### 2.2. Characterization of Catalysts

The surface morphologies of different catalyst samples were tested using an S-4800 SEM instrument (Hitachi High-Technology Co., Ltd., Tokyo, Japan) and transmission electron microscopy (TEM). X-ray diffraction (XRD, D/Max2000, Rigaku, Tokyo, Japan) was investigated through radiation of Cu-Kα. X-ray photoelectron spectroscopy (XPS) was measured using Escalab 250 xi (Thermo Scientific, Loughborough, UK), which provided a base pressure of 5 × 10^−9^ Torr radiated from monochromatic Al Kα (1486.6 eV).

### 2.3. Electrochemical Measurements

Hydrogen evolution reaction activity of the as-prepared catalysts were performed in a standard three-electrode electrochemical cell in argon 0.5 M H_2_SO_4_ solution. A carbon rod and a reversible hydrogen electrode were used as a counter electrode and reference electrode, respectively. The work electrode was modified with a catalyst layer by dropping 5 µL of catalyst ink on the glassy carbon electrode (GCE) with a diameter of 3 mm. All the linear sweep voltammetry (LSV) curves were obtained at scanning rate of 5 mV s^−1^. All potentials were calibrated without iR compensation. The frequency of electrochemical impedance spectroscopy (EIS) measurements ranged from 100 to 0.1 Hz at the potential of −0.25 V.

## 3. Results and Discussion

### 3.1. Characterization of Catalysts

**Figure 1:**
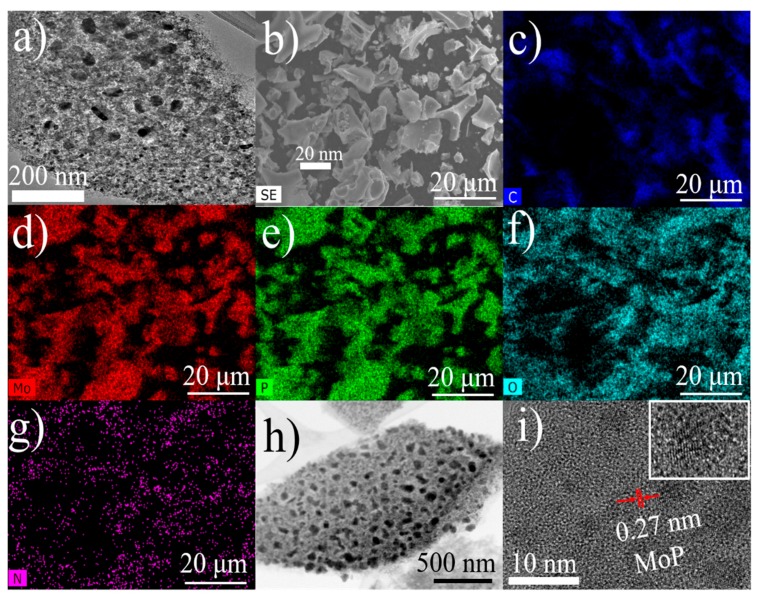
MoP/NPC: (**a**) TEM; (**b**) SEM image; (**c**–**g**) EDS elemental mappings of C, Mo, P, O, and N; (**h**) Scanning transmission electron microscopy (STEM) image; (**i**) HRTEM image (inset: detailed).

The microstructure and morphology of the as-prepared catalysts were investigated using scanning electron microscopy (SEM) and transmission electron microscopy (TEM). As shown in Figure 1a,h and Figure S1, the structure of prepared catalysts were composed by irregular nanoparticles. The catalyst of MoPON/C also presented irregular shape from the energy dispersive spectrometer (EDS) elemental mappings (Figure S2). The serious aggregation of MoPON/C (Figures S3,S4) may have resulted in the catalytic performance decline. Figure 1a shows the TEM image of MoP/NPC, revealing that some small nanoparticles were well dispersed on the carbon layer matrix. The prepared catalyst was mainly composed of C, Mo, P, O, and N (Figure 1c–g, Figure S5). The relatively uniform dispersion could expose abundant active sites and rich channels for the diffusion of electrolytes, which is beneficial for the catalytic process [32,33]. As observed from Figure 1i, a high-resolution transmission electron microscopy (HRTEM) image of a MoP/NPC exhibited that the catalysts were coated by graphitic carbon shells, and the lattice plane distance of MoP was 0.27 nm, which can be ascribed to the lattice plane (101) of MoP [32].

**Figure 2:**
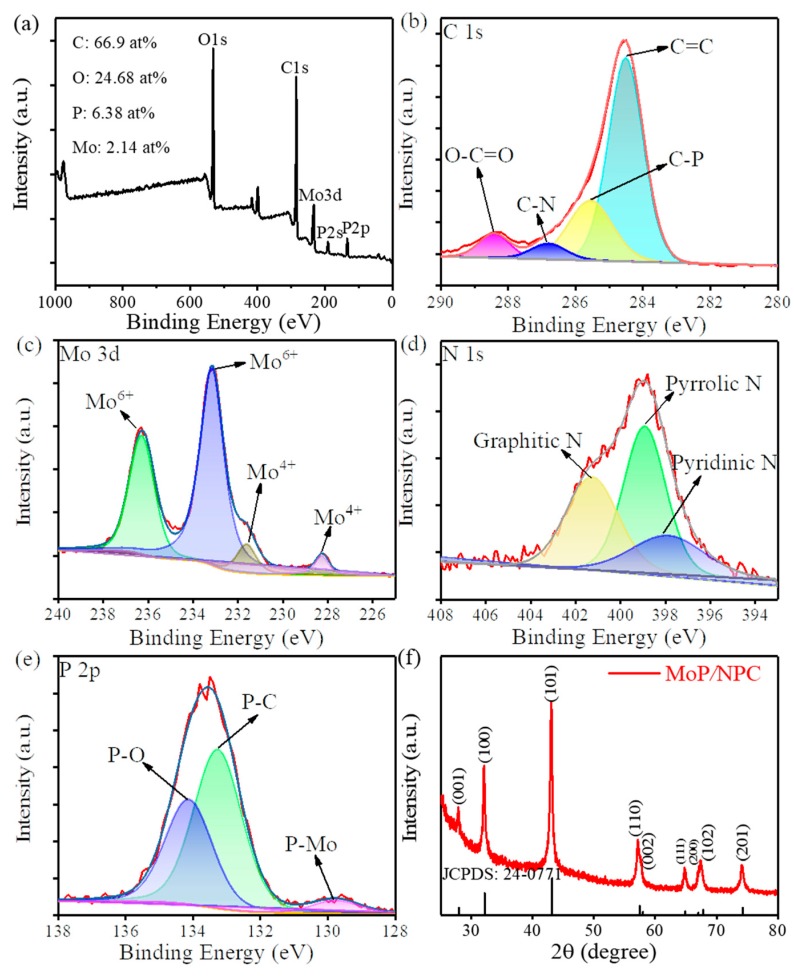
MoP/NPC: (**a**) XPS survey spectrum; (**b**) high-resolution XPS spectra of C 1s; (**c**) Mo 3d; (**d**) N 1s; (**e**) P 2p; and (**f**) XRD pattern.

X-ray photoelectron spectroscopy (XPS) tests were conducted to investigate the composition and chemical valence of the prepared catalysts. In line with the elemental mappings in Figure 1c−f, C, Mo, P, and O were obviously detected in the XPS survey spectrum (Figure 2a). The atomic percentages of C, O, P, and Mo were 66.90, 24.68, 6.38, and 2.14 at. %, respectively. As detected in Figure 2b, four different peaks at 284.5, 285.5, 286.8, and 288.4 eV can be attributed to C−C, C−P, C−N, and O−C=O, respectively [34,35]. The existence of C−N demonstrates the heteroatom N-doping in the graphitic carbon shells, and the C−P might originate from MoP bonded to the graphitic carbon shell [36]. Typically, the Mo 3d peak can be fitted with four regions in Figure 2c, indicating that there were two valence states (+4, +6) for the Mo element in the MoP/NPCs, which confirms the existence of MoP [27]. The high oxidation state of Mo^4+^ (228.2 and 231.6 eV) and Mo^6+^ (233.2 and 236.3 eV) in MoP/NPCs may be caused by the surface oxidation suffering from air contact [32]. For the high-resolution N 1s spectrum in Figure 2d, three peaks at 398.2 eV, 399.2 eV, and 401.5 eV were assigned to pyridinic N, pyrrolic N, and graphitic N, which was ascribed to the heteroatom N-doping in the graphitic carbon [37]. The peak in the P 2p spectrum (129.7 eV) (Figure 2e) can be assigned to P−Mo which was defined as molybdenum phosphide, and a benefit for the catalytic process. Compared with the high resolution P 2p of MoPON/C shown in Figure S6d, the absence of P−Mo in high-resolution P 2p demonstrates the inexistence of MoP. Consequently, MoP-NPC and MoPON/C exhibited different catalytic performance. The peak located at 133.3 eV is the representation of P bonded to C, further manifesting the connection between MoP and graphitic carbon shell. Another obvious peak at 134.1 eV was ascribed to the surface P−O group, which was attributed to the oxidation of catalysts in the air [27]. X-ray diffraction (XRD) was investigated to further confirm the composition of MoP/NPCs. As depicted in Figure 2f, the peaks located at 27.81°, 32.03°, 43.06°, 57.47°, 57.81°, 64.85°, 67.03°, 67.52°, and 74.21° indexed to (001), (100), (101), (110), (002), (111), (200), (102), and (201) facets of pure MoP (JCPDS, No. 24-0771), respectively [32].

### 3.2. Electrocatalytic Properties of Catalysts

**Figure 3:**
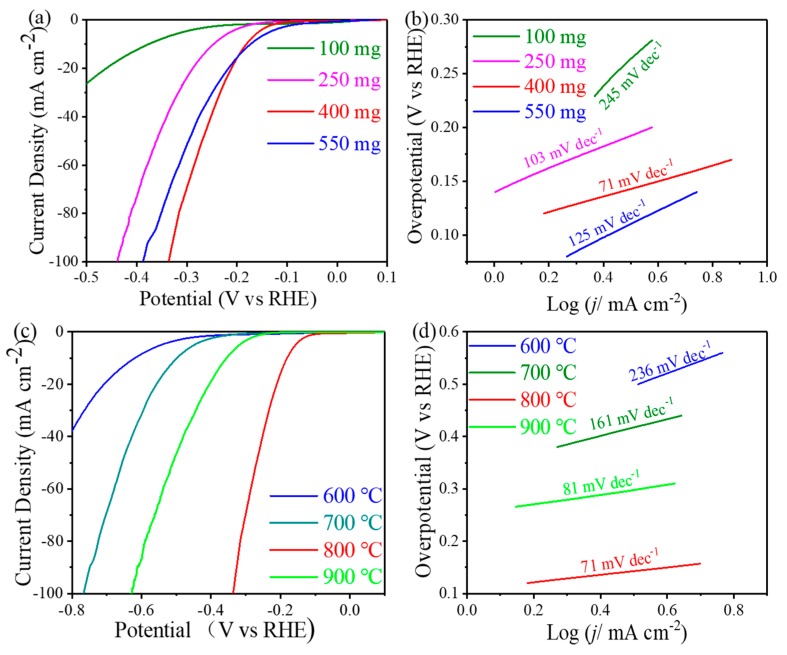
(**a**) Polarization curves of MoP/NPC-100 mg, 250 mg, 400 mg, 550 mg (dosage of ammonium molybdate) in 0.5 M H_2_SO_4_; (**b**) Tafel plots of MoP/NPC-100 mg, 250 mg, 400 mg, 550 mg in 0.5 M H_2_SO_4_; (**c**) polarization curves of MoP/NPC-600 °C, 700 °C, 800 °C, 900 °C in 0.5 M H_2_SO_4_; (**d**) Tafel plots of MoP/NPC-600 °C, 700 °C, 800 °C, 900 °C in 0.5 M H_2_SO_4_.

Hydrogen evolution reaction electrocatalytic properties of different contents were investigated by a standard three-electrode cell in which the carbon rod was used as counter electrode suppressing interruptions of platinum [38]. Figure 3a,c show the HER polarization curves of MoP/NPC, different dosage of ammonium molybdate (100, 250, 400, 550 mg), and various pyrolysis temperatures (600, 700, 800, 900 °C) were determined as objects of study by linear sweep voltammetry (LSV) measurements, respectively. To the best of our knowledge, Tafel slopes are remarkable parameters to suggest HER mechanisms, which is presented in the Tafel Equation η = b log *j* + a (*j* is the current density, b is the slope) [29]. Tafel slopes of MoP/NPC-100, 250, 400, 550 mg and MoP/NPC-600, 700, 800, 900 °C are shown in Figure 3b,d. Tafel slopes of other conditions were all higher than MoP/NPC-400 mg-800 °C (71 mV dec^−1^), the linear portions were consistent with the Tafel equation, revealing the HER proceeds through a Volmer–Heyrovsky mechanism. The smaller Tafel slope for MoP/NPC-400 mg, MoP/NPC-800 °C indicates the better catalytic activity for HER. Overall, the optimal dosage of ammonium molybdate is 400 mg, and pyrolysis temperature is 800 °C.

**Figure 4:**
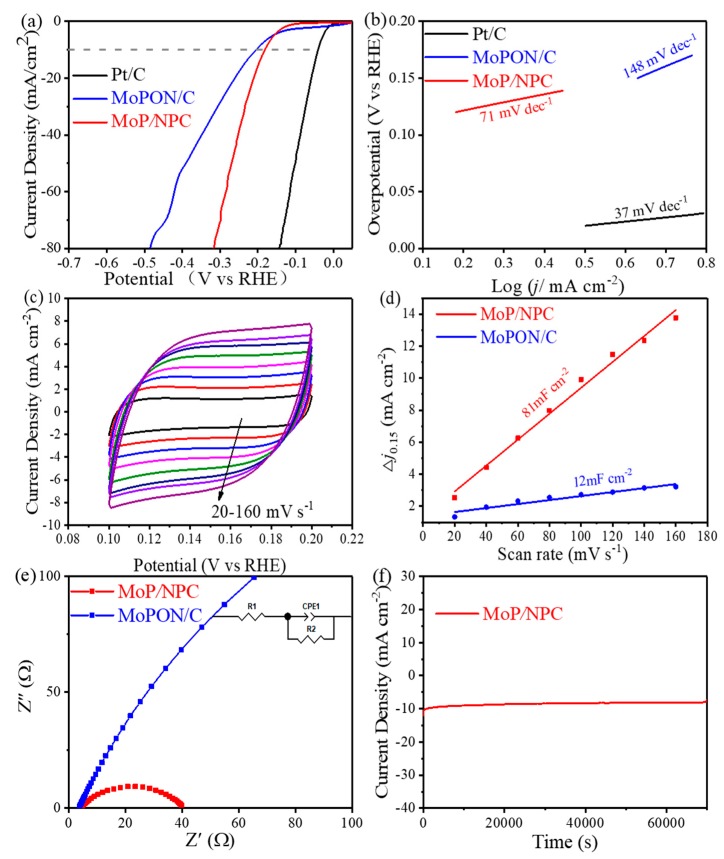
(**a**) Polarization curves of MoPON/C, MoP/NPC, and Pt/C in 0.5 M H_2_SO_4_; (**b**) Tafel plots of MoPON/C, MoP/NPC, and Pt/C in 0.5 M H_2_SO_4_; (**c**) CVs for MoP/NPC with different rates from 20 to 160 mV s^−1^; (**d**) the capacitive currents plotted as a function of scan rate; (**e**) Nyquist plots of electrochemical impedance spectra (EIS) of MoP/NPC and MoPON/C recorded in 0.5 M H_2_SO_4_. Inset: one-time-constant model equivalent circuit used for data fitting of EIS spectra (R1 is the overall series resistance, CPE1 is the constant-phase element and resistance related to surface porosity, R2 is the charge-transfer resistance related to HER process); (**f**) time-dependent current density curve of MoP/NPC under a static overpotential of 0.18 V for 19 h.

Figure 4a shows the HER polarization curves of Pt/C, MoPON/C and MoP/NPC. It is well known that Pt/C with low overpotential and high current density is determined as the superior material for HER [39]. Compared to MoPON/C with an onset potential of 148 mV, the MoP/NPC shows the smaller onset potential of 90 mV (1 mA cm^−2^). Overpotentials of 205 mV is needed to conduct a current density of 10 mA cm^–2^ for MoPON/C, demonstrating the relatively inferior HER activities of this catalyst compared with that of MoP/NPC (180 mV). In addition, the current density of MoP/NPC is higher than that of MoPON/C at the range of potential. To investigate the HER reaction mechanisms of these catalysts, Tafel plots derived from the polarization curves are shown in Figure 4b. The Tafel slope is 71 mV dec^−1^ for MoP/NPC, slightly larger than that of Pt/C (37 mV dec^−1^), confirming the highly efficient kinetics of MoP/NPC for HER, which follows a Volmer−Heyrovsky mechanism [40]. 

The electrochemical active surface area (ECSA) is regarded as a quantitative index for surface roughness. For that, the cyclic voltammograms (CVs) of MoP/NPC and MoPON/C were measured in the region of 0.1–0.2 V (Figure 3c, Figure S7). As demonstrated in Figure 3d, the capacitance of the double layer (C_dl_) for MoP/NPC was 81 mF cm^−2^, which was much higher than MoPON/C (12 mF cm^−2^), indicating a larger ECSA for the MoP/NPC. This phenomenon may be the result of the lattice of MoP, which can expose more electrochemically active sites. Further, electrochemical impedance spectroscopy (EIS), which reflects the charge transfer kinetics [41], were also performed. The Nyquist plots in Figure 4e reveal a small charge transfer resistance for MoP/NPC towards the HER process, suggesting the high intrinsic conductivity and faster Faradaic process in HER kinetics of MoP/NPC. In addition, i–t measurements of MoP/NPC over extended periods were conducted at a constant overpotential of 0.18 V. As demonstrated in Figure 4f, the MoP/NPC exhibited an excellent electrocatalytic stability without obvious current loss within 19 h. In conclusion, these results further indicate that the MoP nanoparticles are the main HER active sites.

## 4. Conclusions

In summary, MoP decorated on NPCs as an efficient electrocatalyst for HER was successfully synthesized via annealing a mixture of ammonium molybdate and phytic acid. The MoP/NPC catalyst delivers highly efficient HER activity with a low onset potential of 90 mV, and a small Tafel slope (71 mV dec^−1^), as well as extraordinary stability in acidic electrolyte. Therefore, this work may open up a new strategy for the design and synthesis of sustainable and effective electrocatalysts as alternatives to non−Pt catalysts for HER.

## Supplementary Materials

The following are available online at http://www.mdpi.com/1996-1944/11/8/1316/s1, Figure S1: (a) TEM of MoP/NPC, Figure S2: EDS spectra of MoP/NPC, Figure S3: SEM of MoPON/C, Figure S4: TEM of MoPON/C; Figure S5: EDS spectra of MoP/NPC, Figure S6: MoPON/C: (a) XPS survey spectrum; (b–e) high-resolution XPS spectra of Mo 3d, C 1s, P 2p, and N 1s, Figure S7: CVs of MoPON/C in the region of 0.10–0.20 V (vs. RHE).
