# Supplementary material for: N,P-Codoped Carbon Layer Coupled with MoP Nanoparticles as an Efficient Electrocatalyst for Hydrogen Evolution Reaction
Source: Materials (Basel). 2018 Jul 30;11(8):1316. doi: 10.3390/ma11081316 (PMC6117796; doi:10.3390/ma11081316)
Supplement: Supplementary file 1 [file materials-11-01316-s001.pdf]

N,P-Codoped Carbon Layer Coupled with MoP Nanoparticles as Efficient Electrocatalyst for Hydrogen Evolution Reaction

Shuai Wang, Jia Wang, Ping Li, Zexing Wu * and Xien Liu*

State Key Laboratory Base of Eco-chemical Engineering, College of Chemistry and Molecular Engineering, Qingdao University of Science & Technology, Qingdao 266042, China; qustwangshuai@qust.edu.cn (S.W.); 18753363755@163.com (J.W.); qustlipingest@163.com (P.L.)

* Correspondence: splswzx@qust.edu.cn (Z.W.); liuxien@qust.edu.cn (X.L.); Tel.: +86-187-6390-9193 (Z.W.); +86-139-6968-0591 (X.L.)

HER in acid electrolyte.

Before electrochemical tests, 60.0 μL of Nafion solution (5.0 wt. %) containing 3.0 mg catalyst was adequately dispersed into the solution of ethanol (0.30 mL) and deionized water (0.15 mL), then the solution was ultrasonicated to form a homogeneous ink. Afterwards, 5.0 μL of obtained catalyst ink (0.23 mg cm^{-2}) was dropped onto electrode surface.

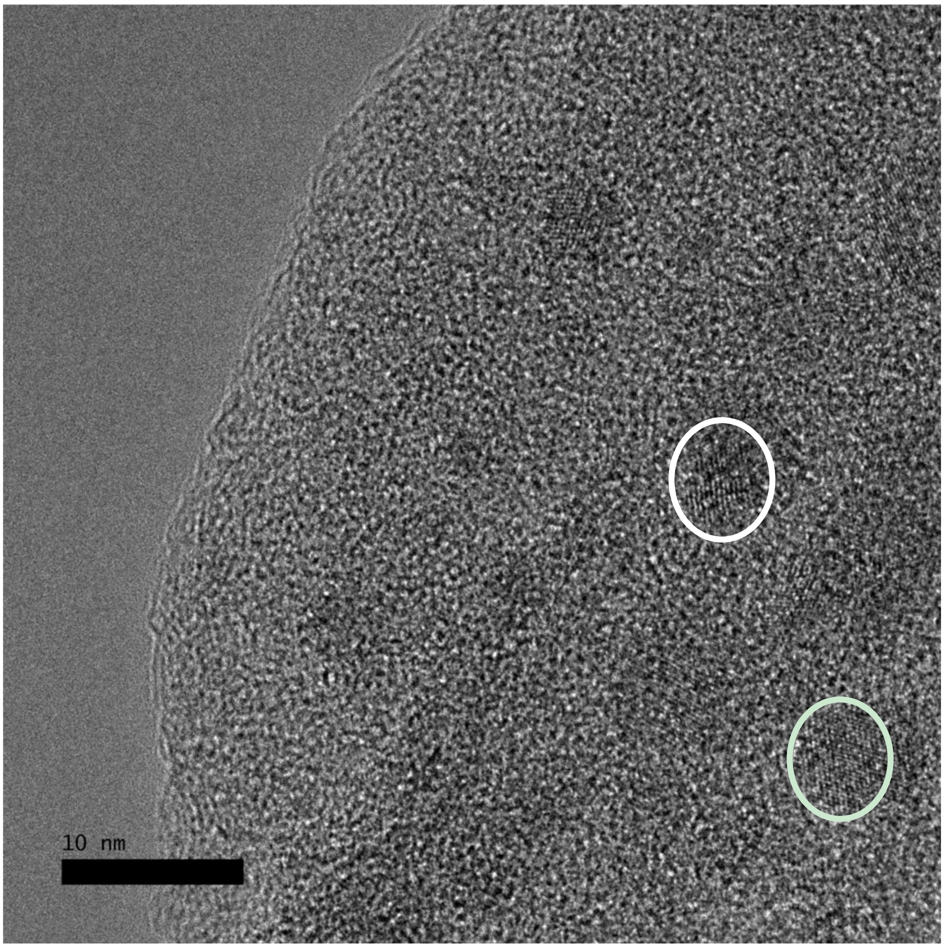

Figure S1. TEM of MoP/NPC.

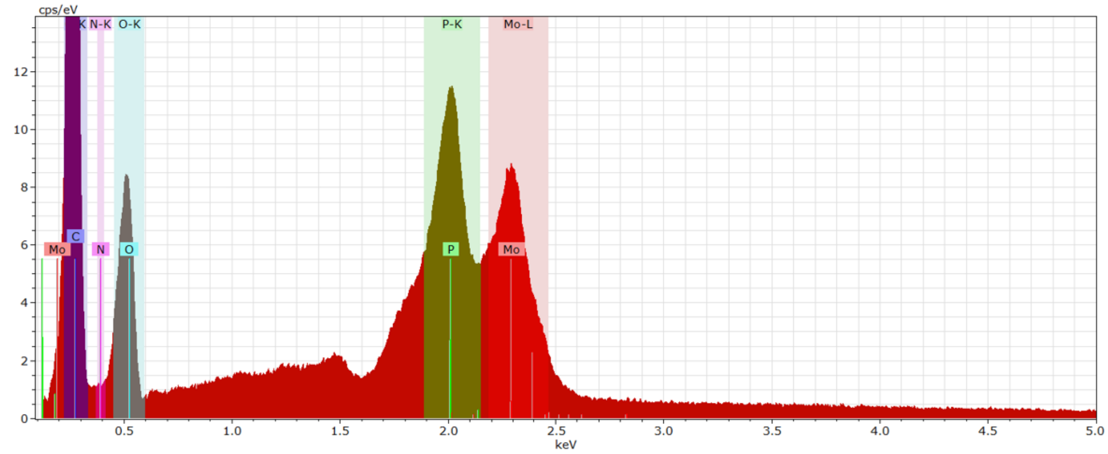

Figure S2. EDS spectra of MoP/NPC.

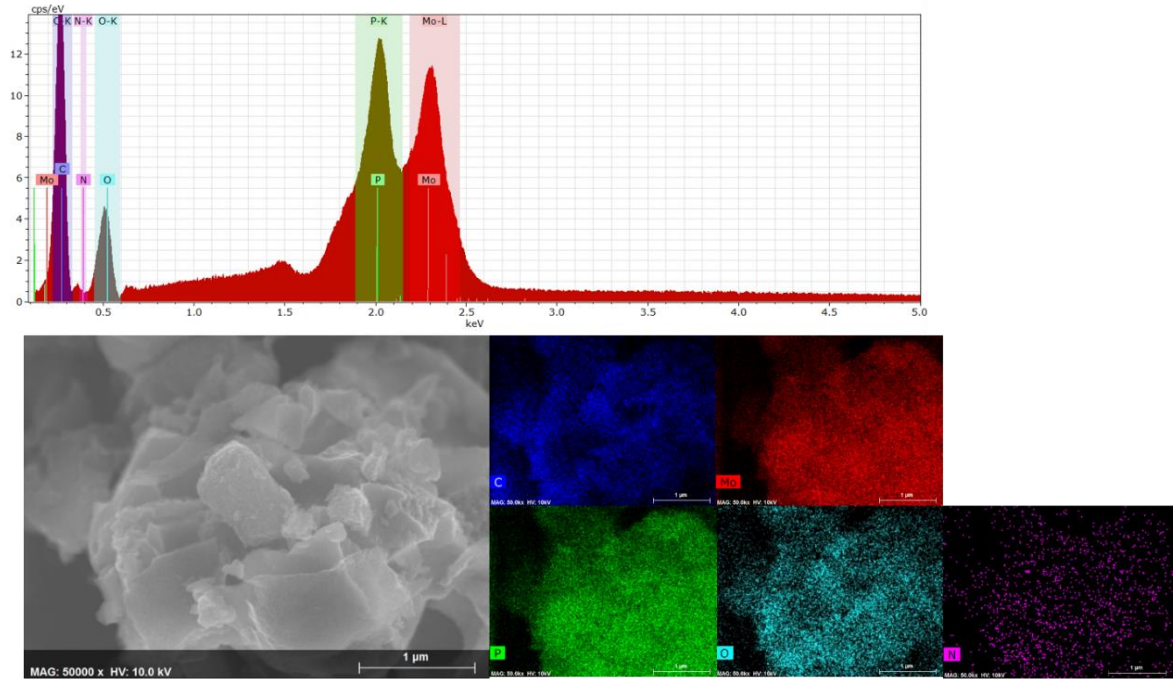

Figure S3. MoPON/C: EDS spectra, SEM and elemental mappings of C, Mo, P, O, N.

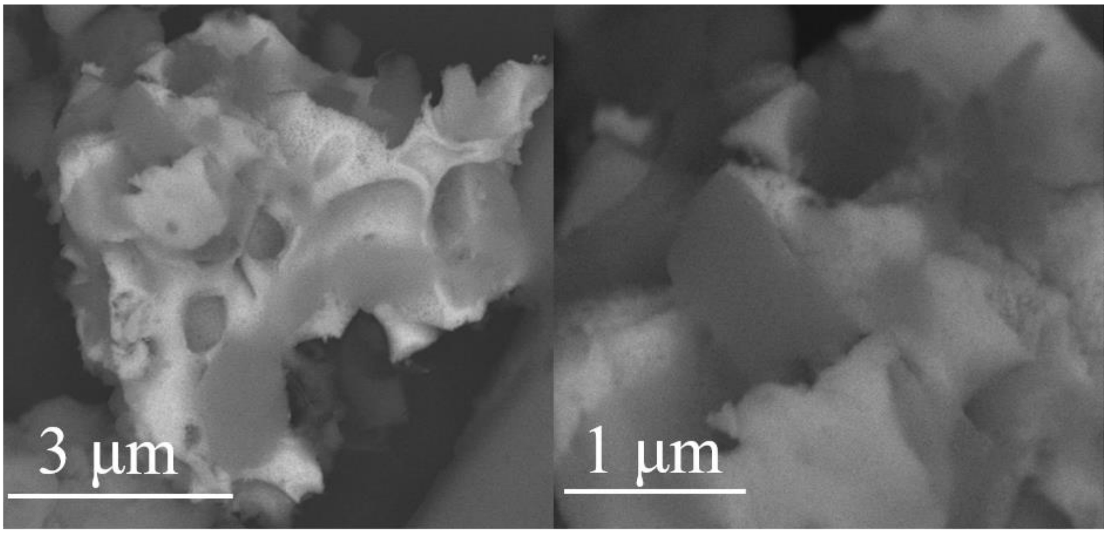

Figure S4. SEM of MoPON/C.

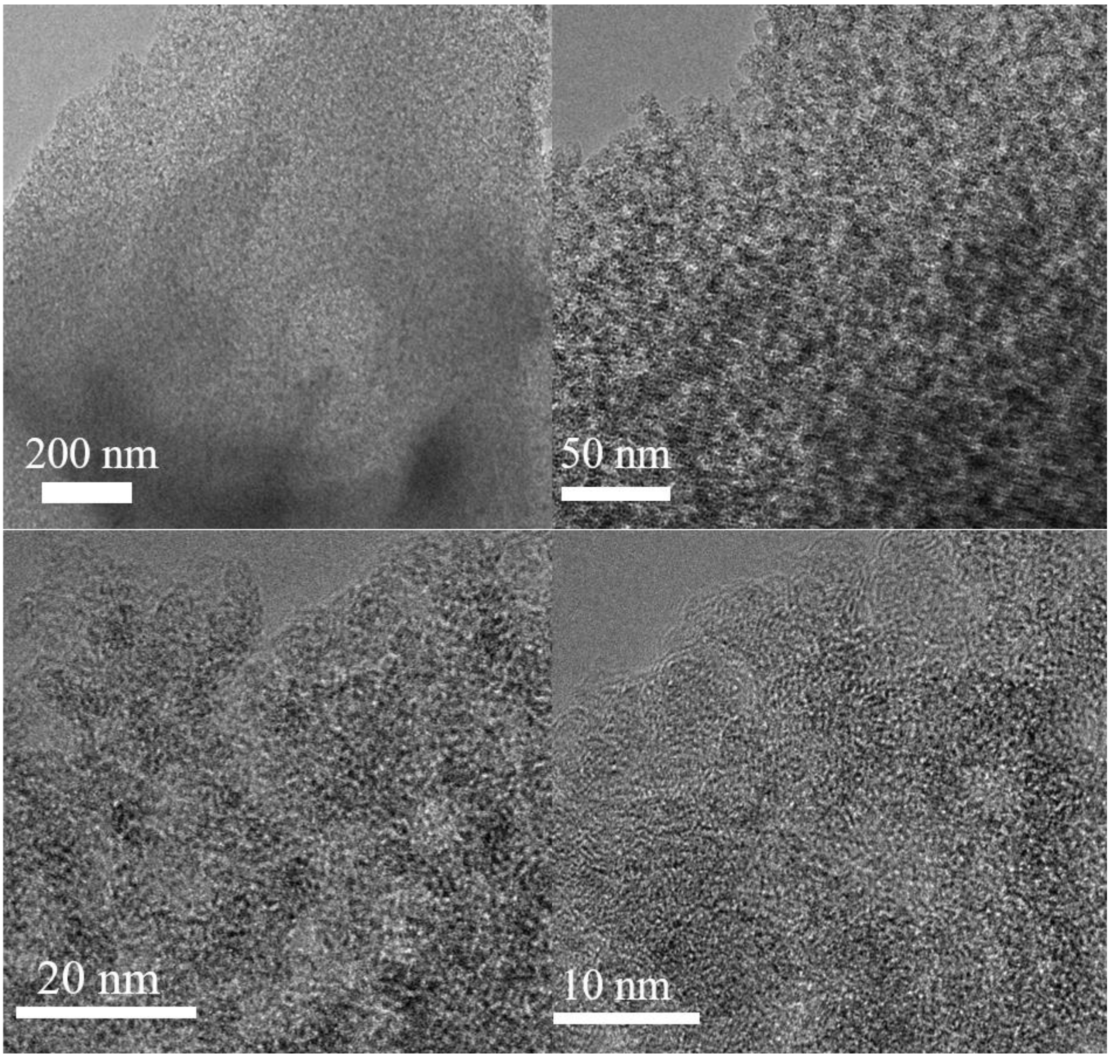

Figure S5. TEM of MoPON/C.

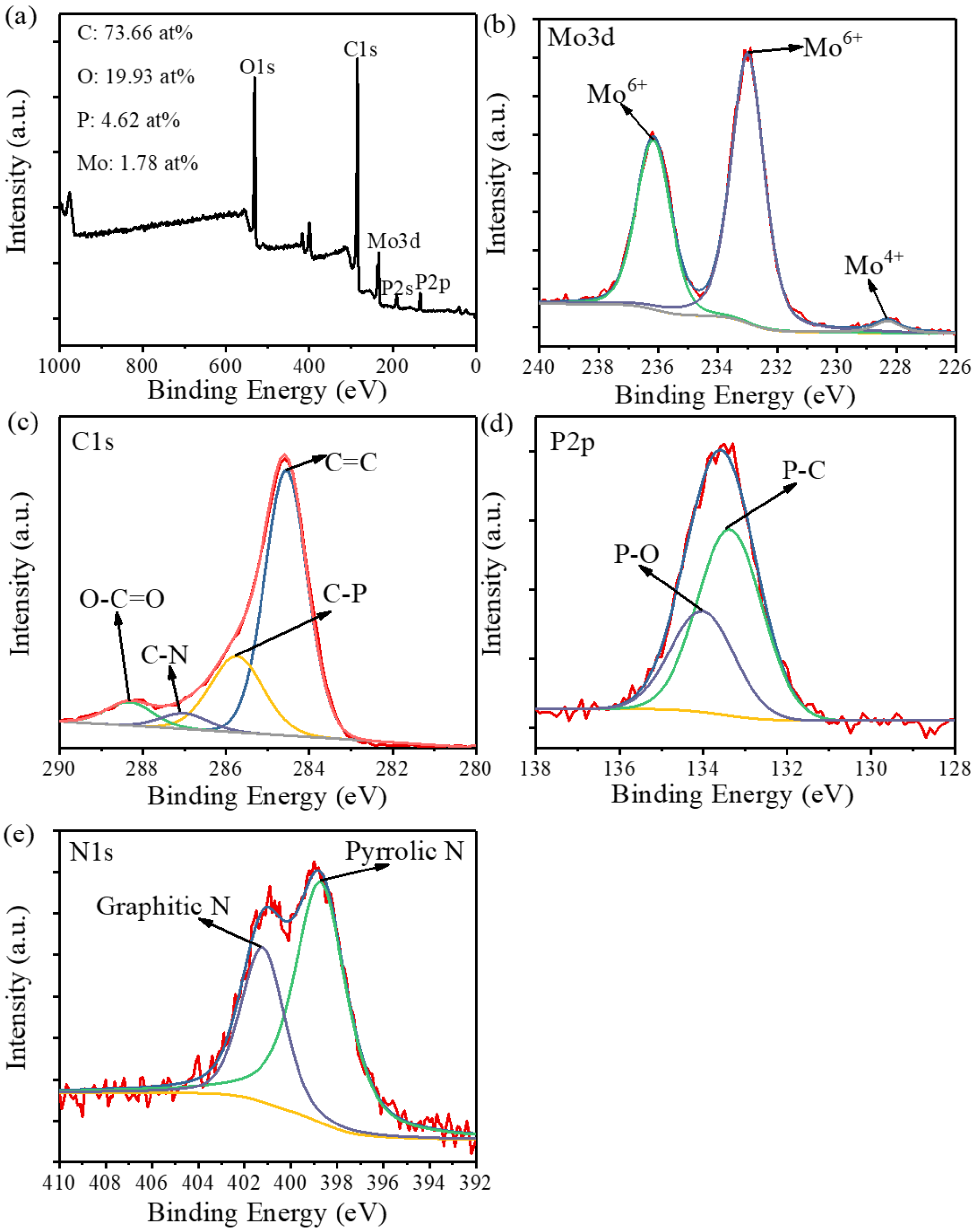

Figure S6. MoPON/C: (**a**) XPS survey spectrum; (**b**–**e**) High-resolution XPS spectra of Mo 3d, C 1s, P 2p and N 1s.

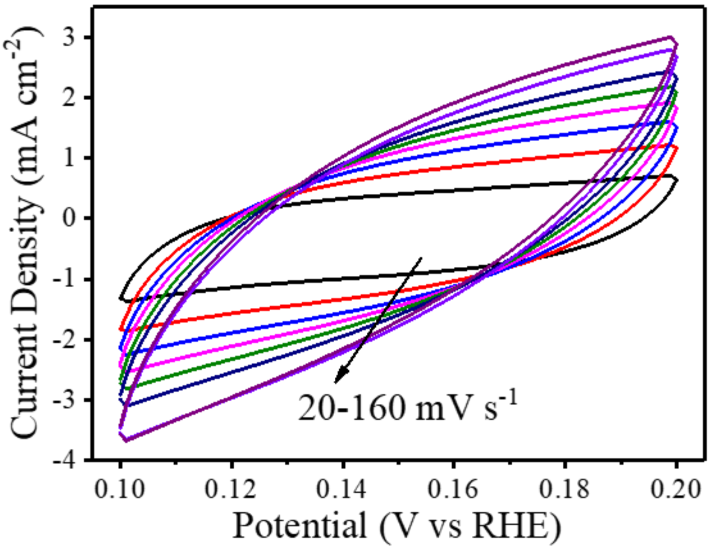

Figure S7. CVs of MoPON/C in the region of 0.10–0.20 V (vs RHE).
